# Increased risks of malaria due to limited residual life of insecticide and outdoor biting *versus* protection by combined use of nets and indoor residual spraying on Bioko Island, Equatorial Guinea

**DOI:** 10.1186/1475-2875-11-242

**Published:** 2012-07-26

**Authors:** John Bradley, Abrahan Matias, Christopher Schwabe, Daniel Vargas, Feliciano Monti, Gloria Nseng, Immo Kleinschmidt

**Affiliations:** 1 Department of Infectious Disease Epidemiology, London School of Hygiene and Tropical Medicine, London, UK; 2 Medical Care Development International, Malabo, Equatorial Guinea; 3 Medical Care Development International, Silver Spring, MD, USA; 4 Ministry of Health and Social Welfare, Malabo, Equatorial Guinea

**Keywords:** Malaria, Insecticide residual life, Indoor residual spraying, Long-lasting insecticidal nets, Outdoor biting, Combined interventions, Serological conversion rate, Vector control, *Plasmodium falciparum*, *Anopheles gambiae*

## Abstract

**Background:**

Malaria is endemic on Bioko Island, Equatorial Guinea, with year-round transmission. In 2004 an intensive malaria control strategy primarily based on indoor residual spraying (IRS) was launched. The limited residual life of IRS poses particular challenges in a setting with year-round transmission, such as Bioko. Recent reports of outdoor biting by *Anopheles gambiae* are an additional cause for concern. In this study, the effect of the short residual life of bendiocarb insecticide and of children spending time outdoors at night, on malaria infection prevalence was examined.

**Methods:**

Data from the 2011 annual malaria indicator survey and from standard WHO cone bioassays were used to examine the relationship between time since IRS, mosquito mortality and prevalence of infection in children. How often children spend time outside at night and the association of this behaviour with malaria infection were also examined.

**Results:**

Prevalence of malaria infection in two to 14 year-olds in 2011 was 18.4%, 21.0% and 28.1% in communities with median time since IRS of three, four and five months respectively. After adjusting for confounders, each extra month since IRS corresponded to an odds ratio (OR) of 1.44 (95% CI 1.15–1.81) for infection prevalence in two to 14 year-olds. Mosquito mortality was 100%, 96%, 81% and 78%, at month 2, 3, 4 and 5 respectively after spraying. Only 4.1% of children spent time outside the night before the survey between the hours of 22.00 and 06.00 and those who did were not at a higher risk of infection (OR 0.87, 95% CI 0.50–1.54). Sleeping under a mosquito net provided additive protection (OR 0.68, 95% CI 0.54–0.86).

**Conclusions:**

The results demonstrate the epidemiological impact of reduced mosquito mortality with time since IRS. The study underscores that in settings of year-round transmission there is a compelling need for longer-lasting IRS insecticides, but that in the interim, high coverage of long-lasting insecticidal nets (LLINs) may ameliorate the loss of effect of current shorter-lasting IRS insecticides. Moreover, continued use of IRS and LLINs for indoor-oriented vector control is warranted given that there is no evidence that spending time outdoors at night increases infection prevalence in children.

## Background

Unprecedented scaling up of indoor residual spraying (IRS) and long-lasting insecticidal nets (LLIN) has led to major successes in the fight against malaria in recent years, with some countries recording more than 50% reductions in the annual number of malaria cases over the past decade [1]. Despite these achievements, the malaria disease burden remains intolerably high, with an estimated 655,000 deaths attributed to the disease globally in 2010.

World Health Organization (WHO) position statements of 2006 and 2007 [2,3] supported the use of IRS and LLIN respectively and recommended their implementation at high coverage for the control and ultimate elimination of malaria. There are currently no guidelines for the simultaneous combined use of both methods together in the same area. Nevertheless, some countries are implementing the two methods in combination in an effort to reduce transmission more rapidly. However, apart from some evidence from observational studies that added protection may be gained from combining IRS and LLINs in the same area [4], prospective randomised trials have yet to show unequivocal evidence supporting such a policy [5].

IRS implementation is based on the premise that the residual active ingredient of the insecticide remains at sufficient concentration on treated interior walls to kill mosquitoes throughout the malaria season, or until the following spray round. The relatively short residual lives of WHOPES-approved products [6] present potential problems in long or year-round transmission seasons. In such circumstances, a possible additional rationale for combining the two control interventions is that LLINs may ameliorate the loss of effect of short-lasting IRS insecticides. In this paper the effect of the limited residual life of IRS on prevalence of infection in children in a setting of year-round transmission and how this may be mitigated by simultaneous provision of LLINs was investigated.

The effectiveness of both IRS and LLINs is based on the assumption that most people are indoors or under an LLIN during the night-time, peak-biting hours of *Anopheles* mosquitoes. Recent reports about outdoor biting behaviour observed in *Anopheles gambiae* raise questions about the future effectiveness of these indoor-oriented vector control methods [7,8]. A recent study on Bioko [8] showed extensive outdoor biting behaviour in *Anopheles gambiae s*.*s*. using human landing collections. In view of this finding the potential impact of being outdoors at night on the effectiveness of indoor vector control strategies as measured by infection prevalence was examined in this study.

### Bioko Island

On Bioko Island, Equatorial Guinea (population 250,000) malaria is endemic with continuous year-round transmission. Before 2004, annual entomological inoculation rates (EIR) of over 250 and 750 infectious bites per person per year by *An*. *gambiae* and *Anopheles funestus* respectively were recorded [9]. In 2004 an intensive malaria control strategy was launched by the Bioko Island Malaria Control Project (BIMCP) funded by the Government of Equatorial Guinea and a consortium of private donors led by Marathon Oil Corporation. There was one round of IRS using the pyrethroid deltamethrin (K-Orthrine WP50, Bayer Crop Sciences, Isando, South Africa) in 2004 but evidence of the knock-down resistance (*kdr*) gene in *An*. *gambiae s*.*s*. [10] led to a switch to carbamate insecticide. Since 2005 two annual rounds of IRS with bendiocarb (FicamTM, Bayer) have been carried out with consistently high coverage [11].

In 2005, intermittent preventative treatment for pregnant women (IPTp), case management using artemisinin-based combination therapy (ACT), improvement in diagnosis through training in microscopy and rapid diagnostic tests (RDT) together with the training of health facility staff were introduced as additional measures.

In 2007, 110,000 PermaNet 2.0 (Vestergaard Frandsen, Lausanne, Switzerland) LLINs were distributed through a mass distribution campaign, assisting householders in hanging one net per sleeping area at an average of three nets per household. Initially very high levels of LLIN ownership and usage were achieved [12], but this decreased over time to as low as 5% of two to 14 year-olds reported to have slept under a treated net the previous night in 2011 (BIMCP unpublished data).

Malaria prevalence in children ages 2–15 years declined from 45% (95% CI 40%–51%) in 2004, before the introduction of intensive malaria control to 20% (95% CI 15%–26%) in 2011. Moderate to severe anaemia (Hg < 8 g/dL) dropped from 15% to 2% over the same period, and all cause under-five mortality fell from 152 per 1,000 births to 55 per 1,000 in the first four years post intervention [12]. The 2004 survey was carried out during March/April whilst the 2011 survey took place in August/September, which may result in some residual confounding since the latter is closer to peak month for rainfall (October). Despite these achievements, prevalence of infection with malarial parasites in children two to four years has remained at about 20% over the past few years (BIMCP unpublished data).

## Methods

### Monitoring

Annual household malaria indicator surveys have been conducted on Bioko since 2004. The data used in this paper are from the survey conducted in 2011 and, for comparison, the 2004 baseline survey. Eighteen sentinel sites were designated on the island and houses were randomly sampled from each site, using lists constructed at each site by enumerating all households, using personal digital assistants (PDAs) equipped with global positioning systems (GPSs). The survey instrument was based on the malaria indicator survey developed by the Roll Back Malaria Monitoring and Evaluation Reference Group [13]. In the 2011 BIMCP survey, a question was added asking whether a child had spent any time outside between 22.00 the previous night and 06.00 on the day of the survey. These times were chosen since a study by Reddy *et al.*[8] found that over 90% of outdoor biting occurred between these hours in the Punta Europa region of Bioko in 2009. The peak biting hours were between 23:00 and 24:00. Sample size was powered to show a change in prevalence from 20% to 17% between years, assuming a design effect of 2.5. Details of the annual BIMCP household surveys have been reported previously [11,12,14-16]. Subject to informed written consent from a caregiver, children two to 14 years old had their haemoglobin measured (HemoCue, Ängelholm, Sweden) and were tested for *Plasmodium falciparum* using RDT (ICT™ Malaria Combo Cassette Test ML02, R&R, Cape Town, South Africa). Children testing positive for parasitaemia, with haemoglobin <11 g/dL, or who were febrile, were referred to a local field clinic for appropriate treatment (anti-malarial, anti-pyretic, or iron supplementation).

Data on insecticide residual efficacy were obtained from WHO standard cone bioassays [17] performed on sprayed walls two months, three months, four months and five months after spraying using susceptible adult mosquitoes reared in a local insectary.

### Statistical analysis

Some of the sentinel sites were large and contained several distinct settlements. Since prevalence of malaria infection and delivery of the interventions could vary between communities in the same sentinel site, the latitude and longitude of each household were used to group houses into separate clusters such that the distance between clusters was at least 1 km. These clusters were used for calculating the following cluster level variables: prevalence of infection, median reported time since last spray, and the proportion of children both sleeping under a net the previous night and living in a sprayed house. Clusters containing fewer than five houses were excluded from analysis. Clusters which were part of the city of Malabo were classified as urban, the others as rural. Household socio-economic status (SES) was calculated using the first principal component score based on variables related to household size, asset ownership, livestock ownership and household utilities. The SES scores were converted to quintiles for analysis.

In the annual malaria indicator survey in 2008, filter paper blood samples were collected from 7,387 individuals of all age groups, for detection of antibodies to *P*. *falciparum* Apical Membrane Antigen-1 (AMA-1) by enzyme linked immunosorbent assay (ELISA), for calculating sero-positivity prevalence by age and for estimating the serological conversion rate (SCR) for each sentinel site [18]. SCR has been shown to be a reliable marker of the intensity of malaria transmission [19,20]. In this study, the previously estimated SCR of each sentinel site was used as a proxy for the site-specific, underlying transmission intensity.

Logistic regression was used to estimate unadjusted odds ratios for malarial infection of children aged two to 14 years in relation to the following variables: the median reported time since spray for the cluster, whether the child spent time outside the house the previous night, whether the area was urban or rural, the quartile of SCR of the sentinel site, the quintile of SES of the household, the age of the child grouped as two to four, five to seven, eight to eleven and twelve to fourteen years old, and whether the child slept under a net the previous night.

A second logistic regression was used to estimate adjusted odds ratios. Median reported time since spray for the cluster and whether the child slept under a net the previous night were included in the model as the exposures of interest; the SCR quartile of the sentinel site, the SES quintile of the household and the age of the child grouped as above were included *a priori* as potential confounders. The child spending time outside the previous night and whether the area was urban or rural were not included in the model because they showed no evidence of association with malaria infection and did not confound the association between malaria infection and the exposures of interest.

Standard errors in both analyses were adjusted to account for the survey design by taking into account the between site variation in prevalence using the svy set of commands in Stata. The primary sampling unit (PSU) was set to be the cluster [21].

To investigate the potential effect of combined vector control on infection prevalence, a linear regression was performed with infection prevalence of the clusters as the dependent variable and the percentage of children who both slept under a net the previous night and live in a sprayed house as an explanatory variable of interest and the SCR of the sentinel site as a second explanatory variable to control for the underlying site-specific transmission intensity. Clusters with fewer than 10 children were excluded from this analysis.

All analysis was done using Stata version 12 [22].

### Ethics and informed consent

Ethics approval for the study was granted by the Equatorial Guinea Ministry of Health and Social Welfare and the London School of Hygiene and Tropical Medicine (approval number 5556). Informed written consent was given by each participant or, in the case of children, a responsible adult. In the case of participants being unable to read the text was read, and explained to them and consent was confirmed by an independent witness identified on the consent form.

## Results

**Figure 1:**
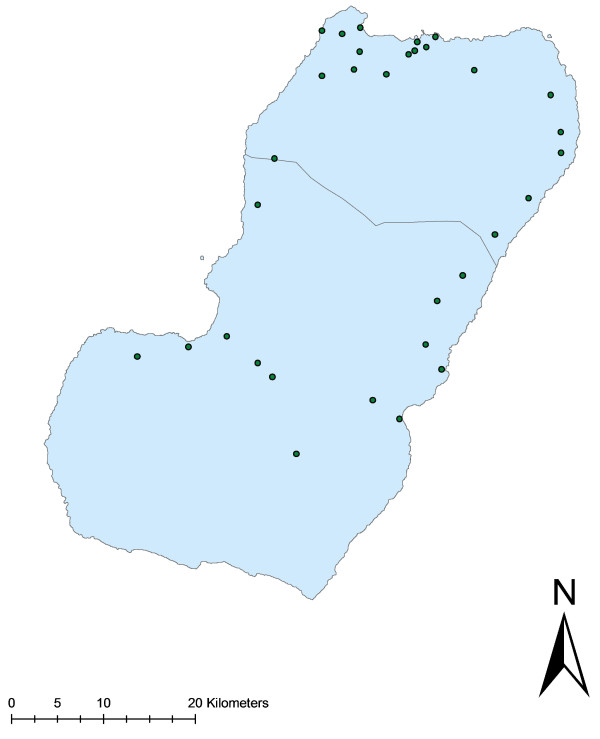
Map of Bioko Island showing the positions of the 32 clusters of houses.

A total of 5,422 children aged two to 14 years of age from 2,337 households were tested for *P*. *falciparum* malaria infection in the 2011 survey. Of these records, 1,428 were excluded from the analysis because the house did not have its co-ordinates recorded and could therefore not be assigned to a cluster; a further 55 records were excluded because they fell in clusters with data on fewer than five houses. The analysis was carried out using the data of the remaining 3,909 children from 1,703 households. Overall prevalence of infection in this sample was 21.0% (819/3909, 95% CI [16.4, 26.4]). Table1 shows that the subset of children retained in the analysis are similar to the complete set with respect to demographic and malaria-related characteristics. The 32 clusters of households used in the analysis are shown in Figure1. The cluster size ranged from five to 318 households. The number of children tested within a cluster ranged from four to 596. The lowest observed infection prevalence in a cluster was 0% (0/18) and the highest was 83.3% (10/12).

**Table 1:** Characteristics of the children used in the analysis and all children tested in the survey

	**Children used in analysis**	**Children tested in survey**
Number of children	3909	5422
Prevalence of malaria (%)	21	20.2
Average age (years)	7.1	7.1
From household in highest SES quintile (%)	23.9	24.1
Slept under net (%)	21.9	21.3
From household sprayed in last six months (%)	51.5	50.2

Table2 presents the prevalence of infection in children aged two to 14 years and odds ratios from logistic regression for variables that were analysed for association with infection. There was some evidence of an association with reported time since spray: three months after spraying each additional month corresponded to an odds ratio (OR) of 1.29 for malaria infection (95% CI 1.00–1.66). The proportion of children who spent time outside the house the night before the survey was 4.1% (153/3,742) with no evidence of being at a greater risk of infection (OR = 0.87, 95% CI 0.50–1.54). Use of a bed net was 21.5% (791/3,672) with strong evidence of a protective effect (OR = 0.66, 95% CI 0.49–0.88). SCR of the sentinel site was strongly associated with malaria prevalence. The bottom four SES quintiles had very similar prevalence of infection but the prevalence in the highest quintile was no adjusted tably lower.

**Table 2:** **Association between infection with*Plasmodium falciparum*in children and factors related to malaria and demographics**

**Effect of**	**Level**	**Number of 2–14 year-olds**	**Infection Prevalence (%)**	**Odds Ratio [95% CI]**	***p*-value**	**Adjusted Odds Ratio [95% CI]^#^**	***p*-value**
Median time since IRS for houses in cluster	3 months	1483	18.4	1.29* [1.00, 1.66]	0.052	1.44* [1.15, 1.81]	0.0026
	4 months	1921	21				
	5 months	505	28.1				
Child slept under net last night	no	2882	21.3	1	0.006	1	0.0021
	yes	791	15	0.66 [0.49, 0.88]		0.68 [0.54, 0.86]	
Age of child	2 - 4 yrs	1205	17.2	1	<0.001	1	<0.001
	5 - 7 yrs	1004	19.8	1.18 [0.97, 1.43]		1.18 [0.96, 1.47]	
	8 - 11 yrs	800	20.9	1.26 [1.00, 1.59]		1.23 [0.94, 1.60]	
	12 - 14 yrs	900	27.1	1.77 [1.49, 2.10]		1.87 [1.49, 2.35]	
Child spent time outside last night	no	3589	21.1	1	0.63		
	yes	153	19	0.87 [0.50, 1.54]			
Cluster setting	urban	2451	19.3	1	0.34		
	rural	1458	23.7	0.77 [0.44, 1.34]			
SES quintile of household	1	545	22.2	1	0.023	1	0.0074
	2	684	26.3	1.25 [0.88, 1.77]		0.99 [0.69, 1.42]	
	3	754	21.1	0.94 [0.59, 1.51]		0.79 [0.50, 1.28]	
	4	735	22.5	1.01 [0.54, 1.88]		0.82 [0.46, 1.46]	
	5	853	15.1	0.62 [0.40, 0.97]		0.49 [0.32, 0.74]	
SCR of sentinel site	Lowest quartile	1034	11.8	1	0.0009	1	0.0004
	2nd quartile	1260	23.1	2.24 [1.10, 4.57]		3.03 [1.81, 5.06]	
	3rd quartile	981	20.3	1.90 [1.13, 3.21]		2.86 [1.88, 4.36]	
	Highest quartile	634	32.7	3.62 [1.99, 6.57]		2.80 1.75, 4.49]	

*for 1 month increase.

^#^ adjusted for median time since IRS for houses in cluster, child slept under net last night, SES quintile of household and SCR of sentinel site.

**Figure 2:**
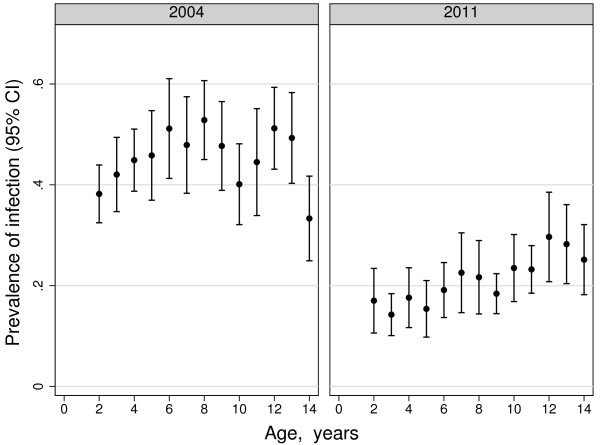
Prevalence of infection in children aged two to 14 years in 2004 and 2011.

There was strong evidence from the 2011 survey that older children were more likely to be infected (Table2). For comparison, Figure2 shows prevalence by age for the 2004 baseline and the 2011 survey. Prevalence of infection was substantially lower in 2011 than in 2004 and the age of peak prevalence shifted from eight year-olds in 2004 to 12 year-olds in 2011 [23].

Table2 shows adjusted odds ratios for median time since IRS for houses in cluster, child slept under net last night, SES quintile of household and SCR of sentinel site using a multivariable logistic regression. There was strong evidence of a protective effect of sleeping under a net (OR = 0.68, 95% CI 0.54–0.86), and an increased risk associated with longer median time since last spray (OR = 1.44, 95% CI 1.15–1.81). The associations between infection and the potential confounders are similar to those in the unadjusted analysis.

**Figure 3:**
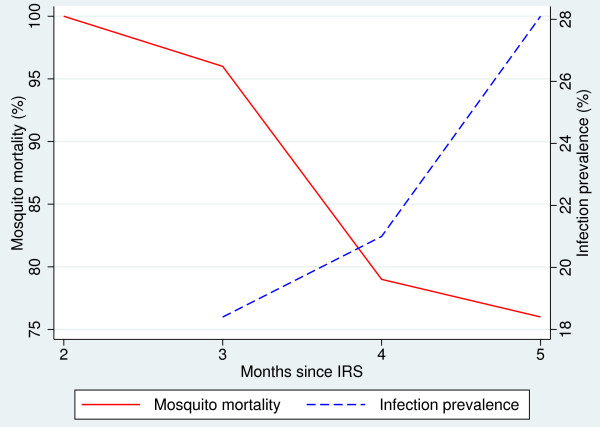
Mosquito mortality and prevalence of infection in children 2 to 14 years against time since last indoor residual spraying.

Table3 shows results from bioassays carried out in 2010 and 2011. In both years mortality decreased rapidly after three months, with 100% mortality after two months dropping to 78% after five months in 2011. Figure3 shows the decline in mosquito mortality in 2011 compared with the rise in infection prevalence in children with respect to time since last spray.

**Table 3:** 24-hour mortality of mosquitoes two, three, four and five months after bendiocarb indoor residual spraying

**Time since spraying, months**	**24-hour mortality, % (N)**			
	**2010**		**2011**	
	Exposed	Controls	Exposed	Controls
2	100 (80)	5 (40)	100 (150)	5 (40)
3	96 (80)	3 (40)	96 (80)	1 (40)
4	73 (80)	3 (40)	81 (97)	8 (40)
5	69 (80)	3 (40)	78 (80)	5 (40)

**Figure 4:**
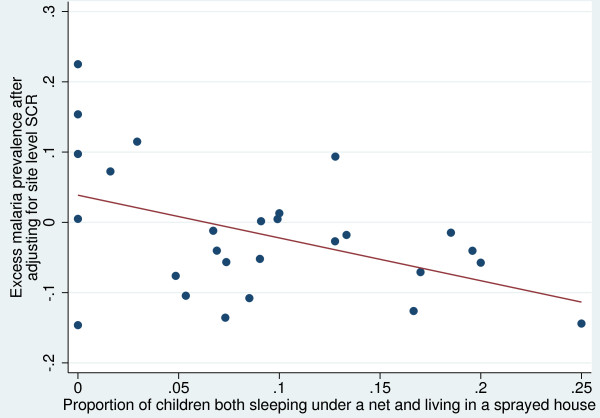
Adjusted prevalence of infection by proportion of children receiving both interventions.

The results of linear regression of the cluster specific prevalence of infection in two to 14 year-old children adjusted for site-specific SCR on the percentage of children who lived in a house sprayed in the last six months and slept under a net the previous night, are shown in Figure4. Three of the clusters were excluded since there were no data on sleeping under nets. A 1% increase of children sleeping in a sprayed house and under a bed net corresponded to a 0.59% decrease in prevalence of malaria infection (95% CI 0.12–1.06, p = 0.016).

## Discussion

This study shows that on Bioko there is a rapid decline of IRS insecticidal effectiveness three months after spraying with bendiocarb. This poses considerable operational challenges since the malaria transmission season is all year round, and indicates that even with two rounds of spraying per year IRS is only partly effective or ineffective for approximately a third of the year. Previous analyses of the residual life of insecticides have been limited to assessing bioassay mosquito mortality at different times post spraying, showing that this varies depending on wall surface substrate, temperature, humidity and other household characteristics [24]. This study confirms the previously reported link between the time since last spraying and infection prevalence [11], and further demonstrates the epidemiological impact of inadequate insecticide residual effect in settings where transmission seasons exceed the residual life of the insecticide. It shows that this epidemiological impact is well correlated with cone bioassay results in which mosquito mortality on walls sprayed with bendiocarb decreases after three months, with 24-hour mortality after five months dropping to as low as 78%.

Therefore, to maximize the impact of IRS, programmes should be conducted in a manner that minimizes the interval between spray rounds that exceeds the effective residual life of the insecticide. It also underscores that in settings of year-round transmission, such as Bioko, there is a compelling need for longer-lasting IRS insecticides.

In addition to confirming the need for longer-lasting IRS insecticides, the present study also shows that the negative effect of the loss in insecticidal efficacy on prevalence of infection may be mitigated by simultaneous high usage of LLINs. Although LLIN usage in Bioko was low in 2011, the analysis shows that individual children sleeping under a net are significantly better protected against malarial infection than those who do not, even after adjusting for SES and site-specific transmission potential (SCR).

Further evidence for the benefit of use of LLINS in addition to IRS on Bioko is given by a more substantial reduction in prevalence of infection relative to the transmission potential in sites where combined use of both IRS and LLINs is high, compared to places with lower coverage of both interventions combined (Figure4). This benefit of using LLINs in addition to IRS may be a result of the short residual life of the insecticide.

Adding a third round of spraying to bridge the gap between spray rounds would be prohibitively costly, excessively demanding on the spray programme, and could lead to non-compliance with householders refusing to have their houses sprayed. On the other hand, ensuring the supply of LLINs in communities that receive IRS can remedy the shortcoming associated with short-lasting IRS insecticides, at least until longer-lasting insecticides of the appropriate insecticide class become readily available. Therefore, despite the high persistent coverage of IRS in Bioko, the national programme should give high priority to scaling up LLIN ownership through a new mass distribution campaign. In addition, the national programme should reinforce health promotion messages on the use of nets even when houses have been sprayed.

In Bioko, as elsewhere, there is concern that with the intensification of vector control interventions that kill *Anopheles* mosquitoes seeking bloodmeals indoors from human hosts during night hours, there may be behavioural selection for increased outdoor biting which could compromise conventional vector control efforts. Outdoor biting has been shown to take place in Bioko in human landing catch observations [8]. The results of the 2011 annual household survey in Bioko show that being outdoors during the night appears to be rare amongst children under 15 years old, and there is no evidence that those who report spending time outdoors at night are at increased risk of infection compared to those who do not. This suggests that outdoor biting, whilst it undoubtedly occurs, is not likely to have a major impact on infection levels among children. Future investigation that includes adults will be necessary to assess whether outdoor biting makes a substantial contribution to transmission in the population as a whole.

The study shows that prevalence of infection is similar across socio-economic groups apart from the wealthiest quintile, who have substantially lower risks of infection. This indicates that the provision of malaria prevention measures are equitable, but that the highest socio-economic quintile is at lower risk, possibly due to better housing, better access to medical care and better health-seeking behaviour.

Infection risk in children under 15 years was shown to increase with age in 2011 (Figure2). Comparison with infection prevalence in 2004, before intensive malaria control was introduced, shows how prevalence has declined in all age groups, but the reduction was most marked in younger children. The shift in peak prevalence of infection to older children is a further indication of sustained reduction in transmission generally over the period from 2004 to 2011 [23].

The results show that prevalence in 2011 was still strongly associated with the sentinel site-specific serological conversion rate. Current site-specific prevalence of infection is a result of both the underlying transmission potential of the site, and the amount of transmission reduction due to effective vector control. The SCR, on the other hand, provides a measure of the inherent transmission potential of each of these sites since it integrates the effects of exposure to plasmodial parasites over a longer period, whereas prevalence of infection is subject to between and within year variation of transmission. For this reason SCR was used as a means of controlling for the baseline transmission potential of a site when assessing the relative impact of interventions as reflected by 2011 infection prevalence.

A limitation of this study was that it was an observational study. Key variables such as time since spraying and spending time outdoors at night were based on recall of respondents, which may be subject to considerable error. The 1428 children who were excluded since their GPS co-ordinates were not recorded reduced the power of the study; however Table1 suggests that excluding them is unlikely to have resulted in bias.

## Conclusion

The results of this study demonstrate the epidemiological impact of reduced mosquito mortality with time since IRS. The study underscores that in settings of year-round transmission there is a compelling need for longer-lasting IRS insecticides, and that high coverage of long-lasting insecticidal nets (LLINs) may mitigate the waning of protective effect conferred by current shorter-lasting IRS insecticides. Moreover, continued use of IRS and LLINs is warranted in the absence of evidence that spending time outdoors at night increases infection prevalence in children.

## Abbreviations

ACT, Artemisinin-based combination therapy; AMA, Apical membrane antigen-1; BIMCP, Bioko island malaria control project; CI, Confidence interval; EIR, Entomological inoculation rate; IPTp, Intermittent preventive treatment in pregnancy; GPS, Global positioning system; IRS, Indoor residual spraying; LLIN, Long-lasting insecticidal net; OR, Odds ratio; PDA, Personal digital assistant; RDP, Rapid diagnostic test; SCR, Serological conversion rate; SES, Socio-economic status; WHO, World Health Organization.

## Competing interests

The authors declare that they have no competing interests.

## Authors’ contributions

Conceived and designed the experiments: IK, CS. Performed the experiments: AM, DV. Analysed the data: JB, IK. Wrote the paper: IK, JB, AM, CS, DV, FM, GN. All authors read and approved the final manuscript.
